# Glucocorticoid receptor occupancy of key bovine alphaherpesvirus 1 (BoHV-1) promoters correlates with chromatin remodeling during reactivation from latency

**DOI:** 10.1128/jvi.00747-25

**Published:** 2025-07-22

**Authors:** Jeffery B. Ostler, Clinton Jones

**Affiliations:** 1 Department of Veterinary Pathobiology, Oklahoma State University, College of Veterinary Medicine 7618, Stillwater, Oklahoma, USA; University of Toronto, Toronto, Ontario, Canada

**Keywords:** chromatin remodeling, reactivation from latency, bovine herpesvirus 1 (BoHV-1), glucocorticoid receptor

## Abstract

Bovine alphaherpesvirus 1 (BoHV-1) continues to be a significant bovine pathogen. Acute infections culminate in lifelong latent infections in sensory neurons of trigeminal ganglia (TG) and the central nervous system. The synthetic corticosteroid dexamethasone consistently initiates BoHV-1 reactivation from latency in calves or rabbits. The immediate early transcription unit 1 (IEtu1) promoter contains two glucocorticoid receptor (GR) response elements and drives the expression of infected cell protein 0 (bICP0) and bICP4. GR, Krüppel-like Factor 15 (KLF15), and dexamethasone treatment cooperatively transactivate the IEtu1 promoter. Conversely, the bICP0 early (E) promoter is cooperatively transactivated by two pioneer transcription factors, GR and KLF4, that are capable of binding silent heterochromatin and activating transcription. Consequently, we hypothesized that GR activates IEtu1 and bICP0 E promoters, which trigger productive infection and reactivation from latency. Notably, BoHV-1 does not replicate in GR null monkey kidney cells (COS-7), unless a GR expression plasmid is transfected with the BoHV-1 genome. Furthermore, GR occupancy of IEtu1 and bICP0 E promoters correlates with viral replication in COS-7 cells. In TG of latently infected calves, IEtu1 and bICP0 E promoters were occupied by a heterochromatin marker H3K9me3 (histone 3 trimethylation at lysine 9), but not GR. Following dexamethasone treatment of latently infected calves for 3 hours, IEtu1 and bICP0 E promoters were occupied by GR and histone 3 acetylated at lysine 9, which correlates with active transcription. Collectively, these studies provide new insights into the mechanism by which stress triggers bICP0 and bICP4 protein expression during reactivation from latency.

**IMPORTANCE:**

Bovine alphaherpesvirus 1 (BoHV-1), a significant pathogen, establishes life-long latency in certain neurons. Dexamethasone, a synthetic corticosteroid, consistently induces BoHV-1 reactivation from latency. Glucocorticoid receptor (GR) and dexamethasone transactivate the immediate early transcription unit 1 (IEtu1) promoter, which drives expression of infected cell protein 0 (bICP0) and bICP4. GR also transactivates the bICP0 early (E) promoter via a ligand-independent manner. Notably, GR and DEX induced BoHV-1 replication in non-permissive COS-7 cells. Furthermore, GR and a histone 3 marker, H3K9 acetylation, are associated with active chromatin and occupy the IEtu1 and bICP0 E promoters when latently infected calves are treated with dexamethasone for 3 hours. Conversely, a heterochromatin marker, histone 3 trimethylated at lysine 9, but not GR, occupied these viral promoters during latency. These studies revealed that GR and dexamethasone play crucial roles in chromatin remodeling of IEtu1 and bICP0 E promoters, which correlate with viral replication and reactivation from latency.

## INTRODUCTION

Bovine alphaherpesvirus 1 (BoHV-1) infection causes upper respiratory tract disease by eroding mucosal surfaces (1) that enhances colonization of *Mannheimia haemolytica* (MH) in the lower respiratory tract (2), which increases the incidence of pneumonia. BoHV-1 also impairs CD8 +T cell recognition of infected cells and CD4 +T cell functions (3). Infection of calves with BoHV-1 and *MH* consistently leads to life-threatening pneumonia (4, 5). Hence, BoHV-1 is a cofactor in certain cases of bovine respiratory disease complex, a poly-microbial disease, reviewed in (6). BoHV-1, including modified live vaccines, is also the most frequently diagnosed cause of viral abortions in North American cattle (7, 8).

**Fig 1:**
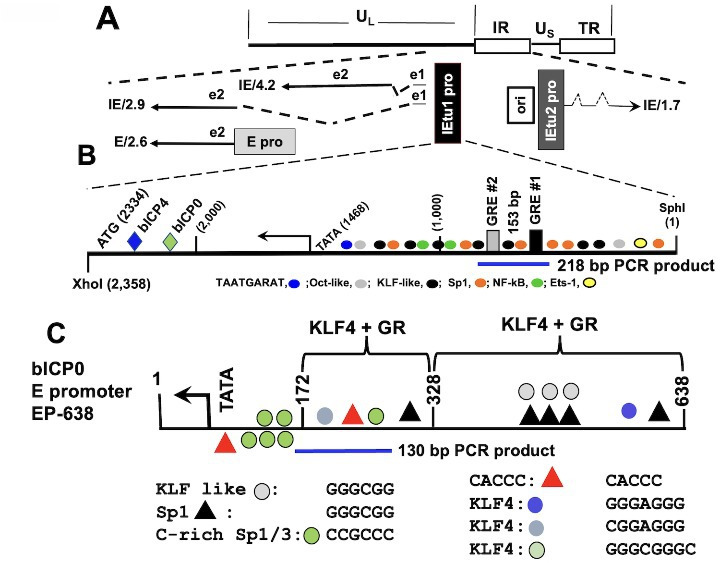
Schematic of the BoHV-1 genome and IEtu1. Panel A: BoHV-1 genome and location of the unique long (U_L_) region, direct repeats (open rectangles), and unique short region (U_S_). IE/4.2 mRNA encodes the bICP4 protein, and IE/2.9 mRNA encodes the bICP0 protein. An IE promoter activates the expression of IE/4.2 and IE/2.9 and is designated IEtu1 (black rectangle) (7, 8). E/2.6 is the early bICP0 mRNA, and its expression is driven by the bICP0 E promoter (E pro; gray rectangle). bICP0 protein coding sequences are in Exon 2 (E2). An origin of replication (ORI) separates IEtu1 from IEtu2. IEtu2 promoter (IEtu2 pro) drives IE1.7 mRNA expression, which is translated into the bICP22 protein. Solid lines in IE/2.9, IE/4.2, and IE/1.7 are exons (e1, e2, or e3), and dashed lines are introns. Panel B: Full-length IEtu1 promoter showing the start site of transcription (arrow), TATA box, binding site for VP16/Oct1 complex (TAATGARAT), and location of GRE#1 plus GRE#2. Additional transcription factor-binding sites in the IEtu1 promoter are denoted. ETs-1 belongs to the ETS (erythroblast transformation-specific) transcription factor family. The alternative bICP4 (blue triangle) and bICP0 mRNA start sites are upstream of the initiating methionine. Numbers in parenthesis are relative to the 5’ nucleotide of the Sph1 restriction site, which is denoted as 1. Location of the 218 bp PCR product is denoted by the blue line, and primers are described in the Materials and Methods section. Panel C: the bICP0 E promoter 638 fragment. The position of the TATA box is shown with an arrow to denote the transcription start site. The positions of putative transcription factor binding sites are shown. The location of the 130 bp PCR product is denoted by a blue line, and primers are described in the Materials and Methods section.

High levels of virus production occur during acute BoHV-1 infection, which triggers apoptosis and inflammation, reviewed in (1). Viral gene expression occurs in three well-defined phases: immediate early (IE), early (E), and late (L). IE transcription unit 1 (IEtu1) encodes two transcriptional regulatory proteins (bICP0 and bICP4), which stimulate productive infection (9–11). IEtu2 encodes the bICP22 protein (10). bICP0 and bICP4 organization is unique for the BoHV-1 genome when compared to other alphaherpesviruses. Instead of repeat sequences flanking both the unique long and unique short regions, as in HSV-1, BoHV-1 only contains repeats between the unique short (U_S_) (Fig. 1A). Intact bICP4 and bICP27 genes are present in the internal repeat (IR) and terminal repeat (TR). Conversely, the IEtu1 transcript extends into the unique long (U_L_): consequently, the bICP0 gene is only present in the genome once.

The IEtu1 promoter, which drives bICP0 and bICP4 expression, contains two consensus GR response elements (GREs) (Fig. 1A and B) and is stimulated by DEX (12, 13). GR stimulates Krüppel-like factor 15 (KLF15) expression, GR and KLF15 interact, and cooperatively transactivate the IEtu1 promoter via a feed-forward transcription loop (13). The bICP0 early (E) promoter (Fig. 1A and C) is synergistically transactivated by GR and Krüppel-like factor 4 (KLF4), both of which are pioneer transcription factors that bind silent chromatin and activate gene expression (14). A tegument protein, VP16, stimulates IE transcription during productive infection by interacting with a TAATGARAT motif present in all IE promoters (15, 16). E mRNAs generally encode nonstructural proteins that promote viral DNA replication. L mRNAs encode proteins that are generally part of the infectious virus.

Sensory neurons in trigeminal ganglia (TG) are primary sites for latency when infection is initiated in the oral, nasal, or ocular cavity (17–20). Lytic cycle viral gene expression and virus production initially occur in acutely infected sensory neurons. Viral gene expression is subsequently extinguished, and significant numbers of infected neurons survive. Surviving infected neurons harbor viral genomes and establish latency. The only viral genes abundantly expressed in latently infected neurons are the latency-related (LR) gene (21) and ORF-E (22), which is upstream of the LR gene. LR gene products promote establishment and maintenance of neuronal latency in calves (23) because these products interact with cellular transcription factors (24–26), impair bICP0 protein expression (27), promote neuronal differentiation (28, 29), and inhibit apoptosis (26, 30). An LR mutant virus with three stop codons at the N-terminus of the first ORF in the LR gene (ORF2) exhibits reduced clinical symptoms because virus shedding from TG, pharyngeal tonsil, and ocular cavity of infected calves is reduced (31). Notably, wild-type (wt) BoHV-1, but not the LR mutant virus, reactivates from latency after DEX treatment, in part because the LR mutant induces high levels of TG apoptosis during establishment of latency (32). ORF2 anti-apoptosis functions enhance establishment and maintenance of latency. LR gene expression is repressed during reactivation from latency (33), implying LR gene products do not directly regulate reactivation from latency.

**Fig 2:**
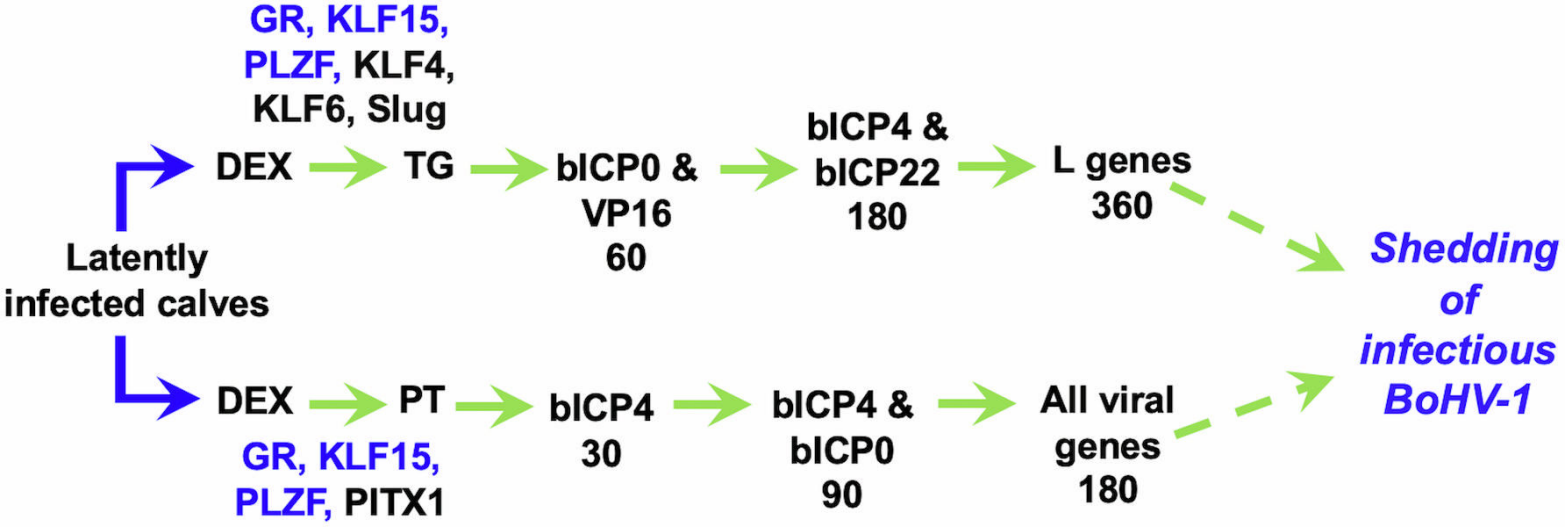
Summary of viral genes and stress-induced cellular transcription factors during reactivation from latency in TG versus pharyngeal tonsil. Time (minutes) denotes when viral RNA is detected in pharyngeal tonsil and viral proteins detected in TG after DEX treatment of latently infected calves. Stress-induced transcription factors in pharyngeal tonsil and TG are blue, and those in black are unique to TG.

In addition to TG, BoHV-1 DNA is consistently detected in pharyngeal tonsil cells of latently infected calves (34). A summary of stress-induced cellular transcription factors identified in pharyngeal tonsil and TG is predicted to be important for reactivation from latency (see Fig. 2 for schematic). Pharyngeal tonsil and TG-specific stress-induced transcription factors may induce distinct patterns of viral gene expression during early stages of reactivation. For example, bICP4 RNA is readily detected in pharyngeal tonsil at 30 minutes after DEX treatment (35). Conversely, bICP0 and VP16 proteins are readily detected in TG neurons within 1 hour after latently infected calves are treated with DEX (36, 37) and then bICP4 and bICP22 (36–38). Based on these observations, distinct cellular factors may trigger bICP4 expression in pharyngeal tonsil. Interestingly, PITX1, a GR-specific coactivator (39), is differentially expressed in pharyngeal tonsil but not in TG during reactivation (40). Differential expression of KLF4 and Slug is detected in TG (40), but not pharyngeal tonsil, during stress-induced reactivation from latency. GR, KLF15, and PLZF are differentially expressed in TG and pharyngeal tonsil during reactivation from latency, suggesting these cellular transcription factors mediate reactivation in TG and pharyngeal tonsil. In sharp contrast to TG, LR RNA is not detected in PT of latently infected calves (35). Since bICP0 and bICP4 are presumed to have equivalent functions as herpes simplex virus 1 (HSV-1) ICP0 and ICP4, we expect bICP4 and bICP0 to play key roles in activating viral gene expression during early stages of BoHV-1 reactivation from latency.

In this study, we initially tested the hypothesis that GR and DEX promote viral replication. Since COS-7 cells do not express GR (41), we compared viral replication in these cells relative to COS-7 cells treated with DEX and/or transfected with a plasmid. Transfection of BoHV-1 DNA into COS-7 cells does not lead to the production of infectious virus. Notably, BoHV-1 virus production was readily detected when COS-7 cells were transfected with BoHV-1 DNA, the GR expression plasmid, and cultures treated with DEX. Hence, this is an intriguing model to identify factors that are essential for BoHV-1 replication and gene expression. Previous studies demonstrated mouse neuroblastoma cells (Neuro-2A) express endogenous GR that associates with BoHV-1 DNA even in the absence of DEX (12, 13). Additional studies compared GR occupancy of key BoHV-1 promoters in TG of a latently infected calf or 3 hours after a single intravenous injection of DEX. Chromatin immunoprecipitation (ChIP) studies demonstrated GR occupies the IEtu1 and bICP0 E promoter in TG during early stages of reactivation but not in TG from latently infected calves. Additional studies revealed histone 3 lysine 9 tri-acetylation (H3K9ac), which is associated with active transcription (42), was detected during reactivation but not during latency. GR was not bound to the IEtu1 or bICP0 E promoters when occupied by histone 3 with trimethylation at lysine 9 (H3K9me3), which is often associated with heterochromatin, reviewed in (43). These studies suggest GR occupancy of the IEtu1 and bICP0 E promoters remodeled chromatin and activated viral gene expression during early stages of reactivation from latency.

## RESULTS

### GR and DEX differentially influence IEtu1 and bICP0 E promoters in COS-7 cells

**Fig 3:**
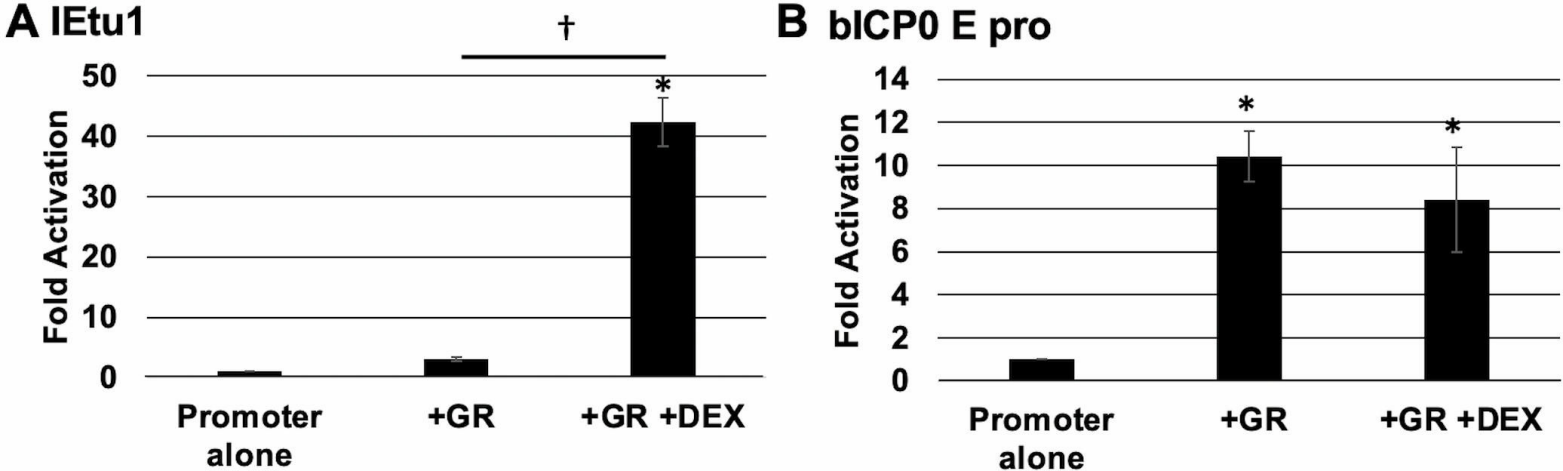
Examination of promoter activity in COS-7 cells. COS-7 cells were cultured in MEM containing 2% charcoal-stripped FBS and transfected with pGL3-promoter constructs containing either IEtu1 (Panel A) or bICP0 E pro (Panel B) promoter sequences cloned upstream of the luciferase gene expression. A plasmid expressing *Renilla* luciferase was cotransfected as a transfection control. Indicated samples were cotransfected with a GR expression plasmid. At 24 hours post-transcription, designated samples were treated with DEX (10 uM). At 48 hours post-transfection, samples were harvested and processed using a Promega dual-luciferase assay kit. Each sample represents the average of three biological replicates, with error bars denoting the standard deviation. Data are presented as fold activation of the indicated sample relative to the promoter construct alone. An asterisk (*) denotes a statistically significant (*P* < 0.01) activation relative to the promoter alone. A statistically significant (*P* < 0.01) difference in promoter activation between two samples is indicated by a dagger (†) over a line connecting the two designated samples. Unless indicated, differences between samples are not significant. Statistical significance was determined by Student’s *t*-test.

Previous studies demonstrated GR and certain stress-induced transcription factors associated with the BoHV-1 IEtu1 or bICP0 E promoters in mouse neuroblastoma cells and bovine kidney cells during transient transfection or productive infection (13, 44–47). Surprisingly, endogenous GR is associated with the BoHV-1 genome in mouse neuroblastoma cells (Neuro-2A), even in the absence of DEX treatment (44). Alternative splicing of GR mRNA leads to GR-α and GR-β, which have multiple isoforms and distinct activities (48, 49). The GR-β isoform is smaller than GR-α, and this truncated GR does not transactivate promoters efficiently (13, 50). All BoHV-1 studies to date focused on the GR-α isoform, referred to as GR hereafter, because it transactivates promoters more efficiently compared to other GR isoforms (49). To eliminate the effects of endogenous GR isoforms, COS-7 cells were used because they do not express GR (41). COS-7 cells transfected with an IEtu1 promoter (Fig. 3A) exhibited negligible promoter activity even when DEX or the GR expression construct was added to cultures. Notably, IEtu1 promoter activity was significantly increased when cells were transfected with the GR construct and cultures treated with DEX, consistent with observations of previous studies (12, 13).

In contrast to the IEtu1 promoter, bICP0 E promoter activity was significantly increased when the GR construct was included in the transfection mixture (Fig. 3B). Addition of DEX to cultures did not increase the effect that GR had on promoter activity, which was consistent with the findings of previous studies that concluded bICP0 E promoter activity was activated by GR via a ligand-independent mechanism (14, 44).

### GR interacts with the BoHV-1 genome in COS-7 cells and is crucial for productive infection

**Fig 4:**
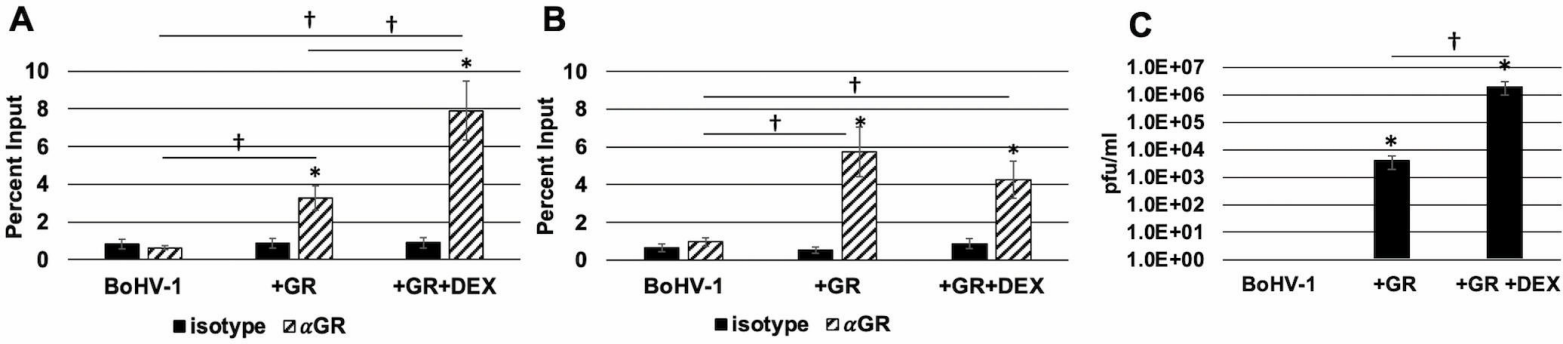
GR occupies the IEtu1 and bICP0 E promoters in transfected COS-7 cells. COS-7 cells were cultured in MEM containing 2% charcoal-stripped FBS and transfected with BoHV-1 genomic DNA. Indicated samples were co-transfected with the GR-α expression plasmid. Empty vector plasmid was added to samples as needed to maintain an equal quantity of DNA across samples. At 24 hours post-transfection, the medium was replaced, and designated samples were treated with 10 uM DEX. At 48 hours post-transfection, cells were formaldehyde-crosslinked to form DNA-protein complexes and harvested for ChIP, as described in the Materials and Methods section. Precipitated DNA was purified and amplified via PCR using the IEtu1 (Panel A) or bICP0 E pro (Panel B) primer sets. PCR products were separated on an agarose gel containing ethidium bromide with bands visualized on a Biorad Chemidoc MP and analyzed using Biorad ImageLab software. The input sample represents 13.3% of the initial total unprocessed lysate, and data for each sample are presented as a percentage of DNA recovered by immunoprecipitation using either the nonspecific isotype IgG or the indicated antibody to the input sample. Each sample represents the average of three biological replicates, with error bars denoting the standard deviation. A statistically significant (*P* < 0.01) enrichment of DNA recovered using the specific antibody relative to isotype is indicated by an asterisk (*). A statistically significant (*P* < 0.01) difference in DNA recovered between two samples is indicated by a dagger (†) over a line connecting the two designated samples. Panel C. Following transfection of COS-7 cells with BoHV-1 DNA, or GR-α expression plasmid or GR-α and DEX, cultures were incubated for 5 days. Media and cells were harvested to determine infectious virus production. Samples were frozen at −80^o^ C and subjected to freeze-thaw (37^o^ to −80°C) for three times. Following the final thawing, infectious virus was identified and measured by titer on MDBK cells. The virus titer is presented as plaque-forming units (pfu) per mL of media. Each sample represents the average of three biological replicates, with error bars denoting the standard deviation. An asterisk (*) denotes a statistically significant (*P* < 0.01) virus titer relative to BoHV-1 DNA alone. A statistically significant (*P* < 0.01) difference in virus titer between two samples is indicated by a dagger (†) over a line connecting the two designated samples. Statistical significance was determined by Student’s *t*-test.

COS-7 cells were transfected with the BoHV-1 genomic DNA alone, a GR expression plasmid, or GR and DEX. The rationale for transfecting viral DNA instead of infecting cells is that the BoHV-1 virion contains three viral transcriptional regulators: VP16, bICP4, and bICP27 (51). ChIP studies were performed on the IEtu1 and bICP0 E promoters (Fig. 1A through C). Significantly higher levels of GR occupied IEtu1 promoter sequences that span the IEtu1 GREs if the GR expression plasmid was included in the transfection when compared with transfecting cells with just BoHV-1 DNA (Fig. 4A). DEX treatment significantly increased GR occupancy of IEtu1 sequences, which was expected because DEX significantly increases GR-dependent IEtu1 promoter activity (12, 13).

Significantly more GR occupied the bICP0 E promoter when transfected with the GR expression construct (Fig. 4B). In contrast to the IEtu1 promoter, GR occupancy of the bCIP0 E promoter was not increased when DEX was added to cultures (Fig. 4B). In fact, DEX reduced GR occupancy; however, the reduction was not significant. Notably, previous studies demonstrated that DEX did not significantly increase bICP0 E promoter activity, suggesting GR-mediated transactivation occurred via a ligand-independent mechanism (14, 44). Although these studies confirmed that GR occupied IEtu1 and bICP0 E promoters, DEX treatment only enhanced GR occupancy on the IEtu1 promoter.

Additional studies tested whether BoHV-1 replicates in COS-7 cells and whether GR and/or DEX influenced replication. Interestingly, infectious virus was not detected in COS-7 cells when transfected with just BoHV-1 DNA (Fig. 4C). In contrast, cotransfection with GR increased virus production approximately 1,000-fold. Virus production increased more than 1,000,000 times when GR was cotransfected and cultures treated with DEX. In summary, these studies revealed DEX significantly increased the GR occupancy of the IEtu1 promoter and significantly increased the IEtu1 promoter activity. Furthermore, viral replication in COS-7 cells required GR or GR and DEX for virus production.

### GR-mediated chromatin remodeling at IEtu1 and bICP0 E promoters in COS-7 cells

**Fig 5:**
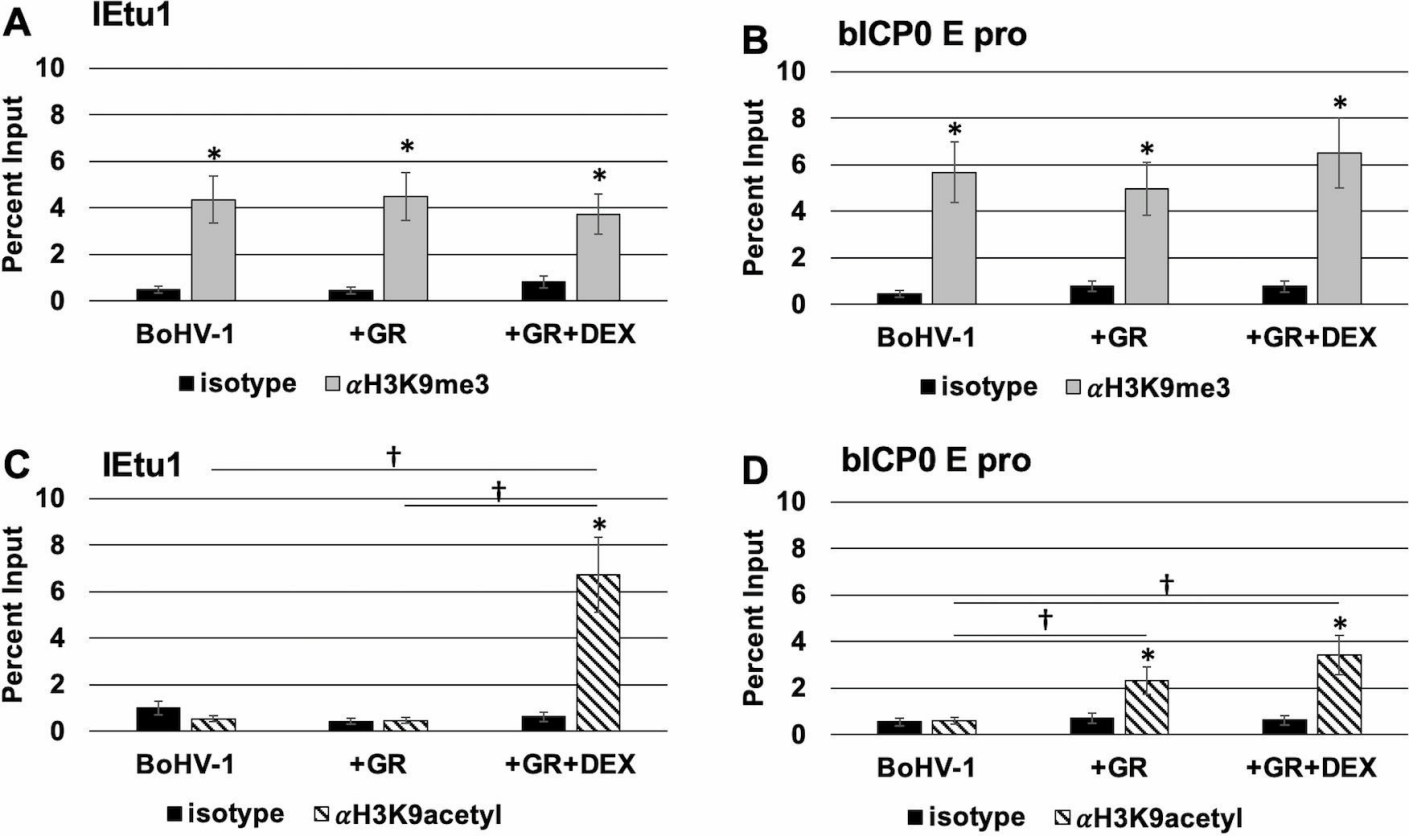
BoHV-1 chromatin modifications in COS-7 cells. COS-7 cells were cultured in MEM containing 2% charcoal-stripped FBS and transfected with BoHV-1 genomic DNA. Indicated samples were cotransfected with a GR-α expression plasmid. The empty vector plasmid was added to samples as needed to maintain an equal quantity of DNA across samples. At 24 hours post-transfection, the medium was replaced, and designated samples were treated with 10 uM DEX in MEM containing 2% FBS. At 48 hours post-transfection, cells were formaldehyde-crosslinked to form DNA-protein complexes and harvested for ChIP. Precipitated DNA was purified and amplified via PCR using the IEtu1 (Panels A & C) or bICP0 E pro (Panels B & D) primer sets. PCR products were separated on an agarose gel containing ethidium bromide with bands visualized on a Biorad Chemidoc MP and analyzed using Biorad ImageLab software. The input sample represents 13.3% of the initial total unprocessed lysate, and data for each sample are presented as a percentage of DNA recovered by immunoprecipitation using either the nonspecific isotype IgG or the indicated antibody relative to the input sample. Each sample represents the average of three biological replicates, with error bars denoting the standard deviation. A statistically significant (*P* < 0.01) enrichment of DNA recovered using the specific antibody relative to isotype is indicated by an asterisk (*). A statistically significant (*P* < 0.01) difference in DNA recovered between two samples is indicated by a dagger (†) over a line connecting the two designated samples. Statistical significance was determined by Student’s *t*-test.

As described in Fig. 4, COS-7 cells were transfected with BoHV-1 genomic DNA alone or in combination with a GR expression plasmid and DEX treatment. ChIP studies were performed using antibodies that specifically recognize histone 3 (H3) lysine 9 trimethylation (H3K9me3) or acetylated H3K9 (H3K9ac). The rationale for examining H3K9me3 is this histone 3 modification is detected on key HSV-1 promoters during establishment of latency (52, 53). Conversely, H3K9ac correlates with active transcription (42). Of note, both modifications are deposited on the same amino acid residue, H3K9, and are mutually exclusive. Any change represents both the removal of one marker and deposition of the other. In COS-7 cells transfected with the BoHV-1 genome, H3K9me3 occupied the IEtu1 promoter (Fig. 5A) and bICP0 E promoter (Fig. 5B), consistent with studies demonstrating H3K9me3 correlates with transcriptionally silent chromatin, reviewed in (54).

Although histone markers for “silent” genes are prominent in viral genomes during latency, histone markers associated with “active” transcription accumulate on key viral promoters during reactivation from latency (42, 52, 53). Cotransfection with GR or GR and DEX treatment did not significantly change H3K9me3 levels that occupy the IEtu1 or bICP0 E promoter. Notably, H3K9ac occupancy of the IEtu1 promoter was significantly increased at the IEtu1 promoter when cotransfected with GR and cultures treated with DEX (Fig. 5C). Occupancy of H3K9ac with the bICP0 E promoter was also significantly increased when GR or GR +DEX was included in the transfection; however, addition of DEX did not significantly increase H3K9ac occupancy (Fig. 5D). Unlike the IEtu1, the bICP0 E promoter does not contain any consensus GREs, and DEX does not increase promoter activation by GR *in vitro*. Furthermore, GR interacts with other transcription factors, including KLF4, to activate this promoter in the absence of DEX. This chromatin remodeling must, therefore, be achieved in a DEX-independent manner. In summary, GR and DEX treatment correlated with occupancy of H3K9ac at the IEtu1 promoter. Notably, GR alone was sufficient for remodeling the bICP0 E promoter in COS-7 cells, as judged by detection of H3K9ac.

### GR occupies the IEtu1 and bICP0 E promoter during DEX-induced reactivation from latency

**Fig 6:**
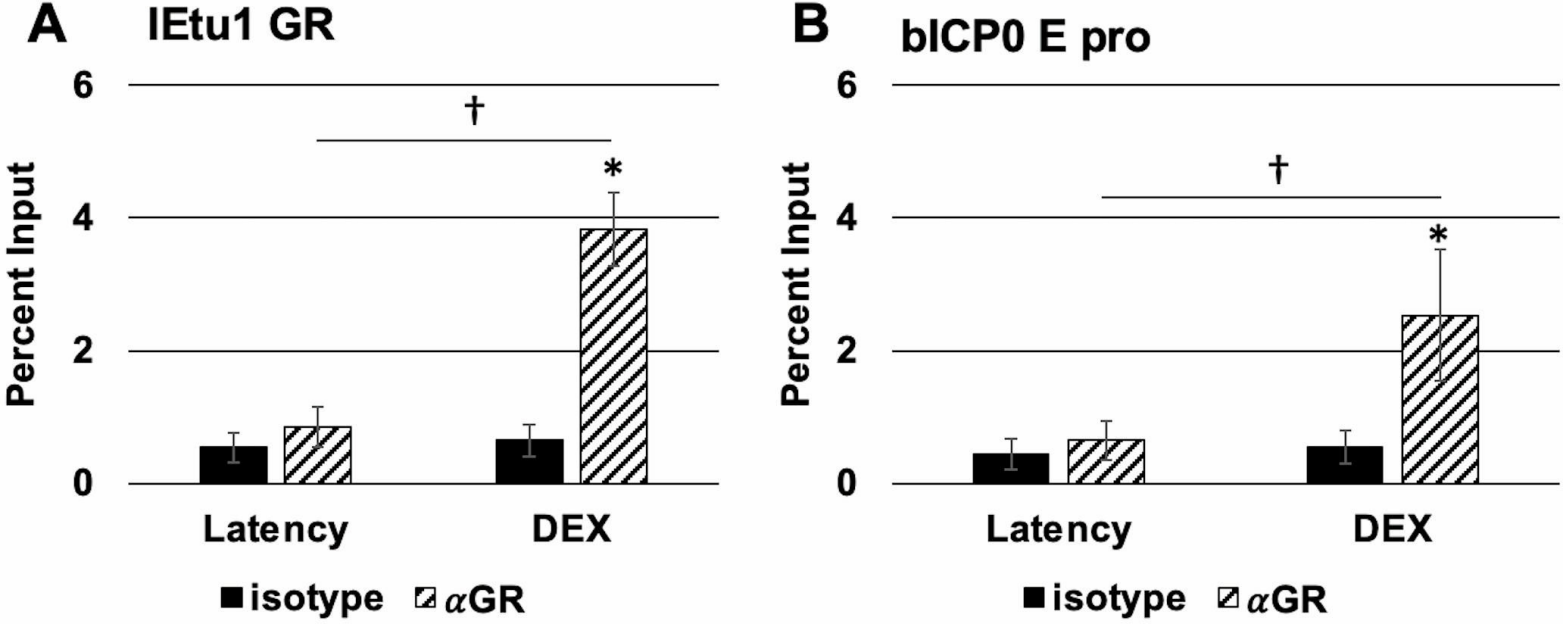
GR occupancy with the BoHV-1 genome during reactivation in calves. TG from a BoHV-1 latently infected calf or latently infected calf treated with DEX for 3 hours was harvested as described in the Materials and Methods section and stored at −80℃. Individual 300 mg samples were finely sliced over dry ice to maintain frozen tissue and formaldehyde-crosslinked to create DNA-protein complexes. The fixed tissue was homogenized by grinding into a fine powder using a liquid nitrogen-cooled mortar and pestle. This powder was suspended in passive lysis buffer and sonicated to produce ~500 nt DNA fragments. Each sample was centrifuged to remove the substantial tissue debris, and the supernatant was collected for processing by ChIP, as described in the Materials and Methods section. Precipitated DNA was purified and amplified via PCR using the IEtu1 (Panel A) or bICP0 E pro (Panel B) primer sets. PCR products were separated on an agarose gel containing ethidium bromide with bands visualized on a Biorad Chemidoc MP and analyzed using Biorad ImageLab software. The input sample represents 13.3% of the initial total unprocessed lysate, and data for each sample are presented as a percentage of DNA recovered by immunoprecipitation using either the nonspecific isotype IgG or the indicated antibody relative to the input sample. Each condition represents the average of three samples taken from TG of a calf, with error bars denoting the standard deviation. A statistically significant (*P* < 0.01) enrichment of DNA recovered using the specific antibody relative to isotype is indicated by an asterisk (*). A statistically significant (*P* < 0.01) difference in DNA recovered from TG of latently infected calves or latently infected calves treated with DEX for 3 hours is indicated by a dagger (†). Statistical significance was determined by Student’s *t*-test.

ChIP studies were performed to test whether GR occupies the IEtu1 and bICP0 E promoters in the TG of a latently infected calf or during early stages after reactivation. Three hours after DEX treatment of latently infected calves was the time point chosen because bICP0 and bICP4 proteins are detected in TG neurons of calves latently infected with BoHV-1 within 3 hours after DEX treatment (36, 37, 38) (Fig. 2 ). GR binding to IEtu1 promoter sequences (Fig. 6A) or bICP0 E promoter (Fig. 6B) in TG of a latently infected calf was not significantly different than that of the isotype control antibody. In contrast, significantly higher levels of GR occupancy were detected at the IEtu1 promoter within 3 hours after an intravenous injection of DEX was given to a latently infected calf. Furthermore, significantly higher GR occupancy was detected at the bICP0 E promoter following DEX treatment because novel proteins in TG neurons must be activated. In summary, these studies revealed GR occupies the IEtu1 and bICP0 E promoters during early stages of DEX-induced reactivation from latency.

### DEX-induced chromatin remodeling at the IEtu1 and bICP0 E promoters during reactivation from latency

**Fig 7:**
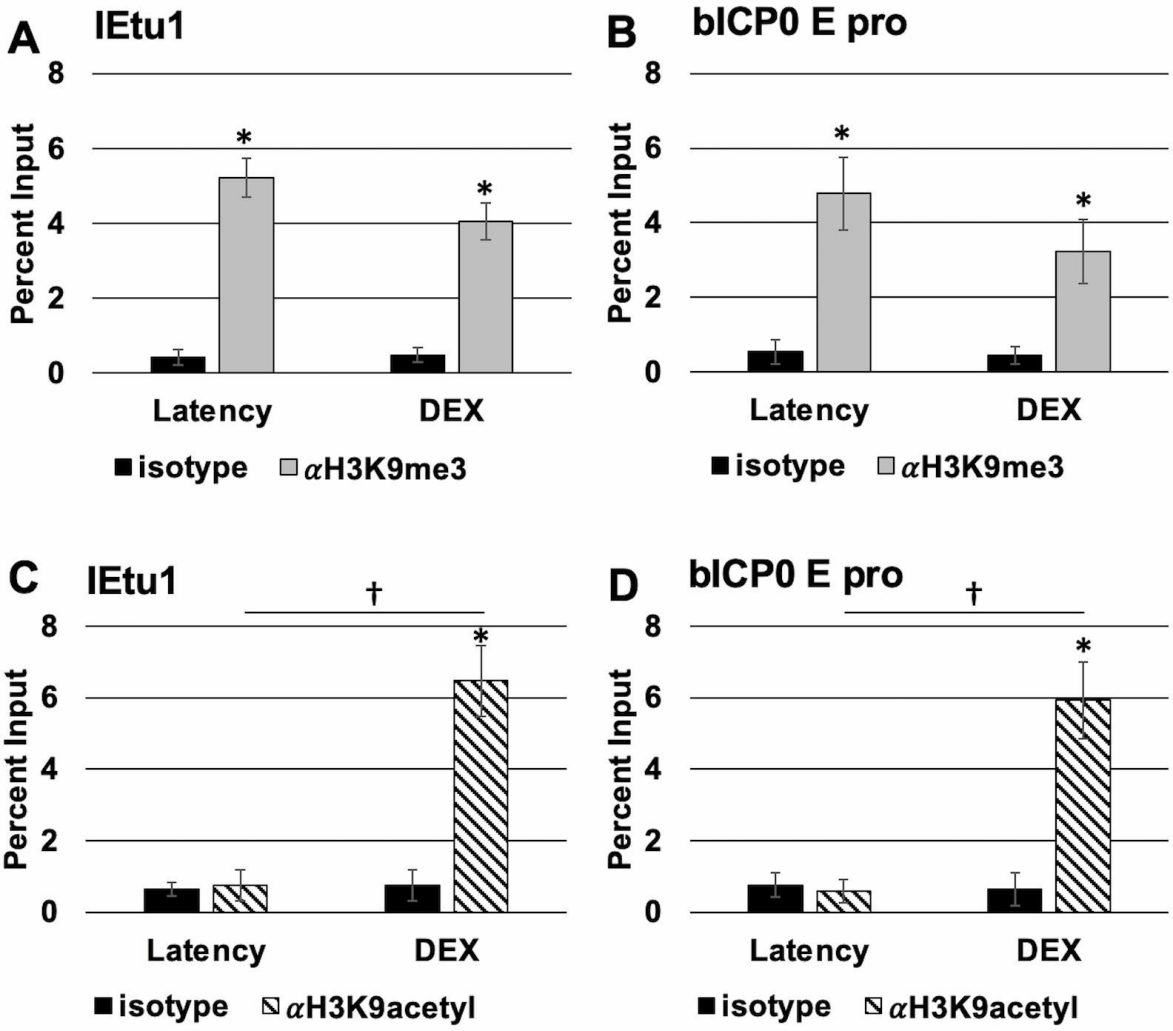
Chromatin modifications in the BoHV-1 genome during reactivation in calves. TG from a BoHV-1 latently infected calf or a latently infected calf treated with DEX for 3 hours was collected as described in the Materials and Methods section and stored at −80°C. Individual 300 mg samples were finely sliced over dry ice to prevent thawing of the tissue and formaldehyde-crosslinked to create DNA-protein complexes. The fixed tissue was homogenized by grinding into a fine powder using a liquid nitrogen-cooled mortar and pestle. The powder was suspended in passive lysis buffer and sonicated to produce ~500 nt DNA fragments. Each sample was centrifuged to remove the substantial tissue debris, and the supernatant was collected for processing by ChIP, as described in the Materials and Methods section. Precipitated DNA was purified and amplified via PCR using the IEtu1 (Panels A and C) or bICP0 E pro (Panels B and D) primer sets. The PCR product was separated on an agarose gel containing ethidium bromide with bands visualized on a Biorad Chemidoc MP and analyzed using Biorad ImageLab software. Input samples represent 13.3% of the initial total unprocessed lysate, and data for each sample are presented as a percentage of DNA recovered by immunoprecipitation using either the nonspecific isotype IgG or the indicated antibody relative to the input sample. Each condition represents the average of three samples taken from TG of a calf, with error bars denoting the standard deviation. A statistically significant (*P* < 0.01) enrichment of DNA recovered using the specific antibody relative to isotype is indicated by an asterisk (*). A statistically significant (*P* < 0.01) difference in DNA recovered from TG of latently infected calves or latently infected calves treated with DEX for 3 hours is indicated by a dagger (†). Statistical significance was determined by Student’s *t*-test.

HSV-1 (52, 53, 55) is organized as heterochromatin during latency, suggesting the BoHV-1 genome is also organized as heterochromatin during latency. To test this prediction, H3K9me3 and H3K9ac were used for ChIP studies of TG from a latently infected calf or a calf treated with DEX for 3 hours to trigger the escape from latency. As expected, H3K9me3 occupied IEtu1 promoter sequences (Fig. 7A) and bICP0 E promoter sequences during latency (Fig. 7B). Following DEX treatment, H3K9me3 was still readily detected, and there was no significant reduction when compared to latency. Conversely, significantly higher levels of H3K9ac occupied the IEtu1 promoter during early stages of DEX-induced reactivation when compared to latency (Fig. 7C). Since these two markers are made on the same amino acid residue, this change correlates with the removal of trimethylation markers and deposition of an acetyl group. The bICP0 E promoter region also revealed H3K9ac occupancy was significantly higher in TG after DEX treatment when compared to TG from latently infected calves (Fig. 7D). In summary, DEX treatment of BoHV-1 latently infected calves led to chromatin remodeling at the IEtu1 and bICP0 E promoters, which correlates with bICP0 and bICP4 protein expressions in TG neurons.

## DISCUSSION

The synthetic corticosteroid DEX consistently induces BoHV-1 reactivation from latency in latently infected calves (56) or rabbits (57, 58). Notably, BoHV-1 is one of the few *⍺-*herpesvirinae subfamily members where reactivation from latency is consistently initiated in the natural host. Previous studies demonstrated GR occupies IEtu1 and bICP0 E promoters in transfected cells and productively infected cells (13, 14, 44, 59). This study revealed that GR, a non-histone protein, occupied IEtu1 and bICP0 E promoters in TG within 3 hours after an intravenous DEX injection was given to latently infected calves. This finding is consistent with those of previous studies demonstrating that bICP0 and bICP4 proteins are detected in TG neurons of latently infected calves within 1.5 to 3 hours after DEX treatment but not prior to DEX treatment (36–38) (Fig. 2). The finding that BoHV-1 replicated in COS-7 cells only when GR and DEX were present highlights the crucial role GR and stress play in BoHV-1 reactivation from latency. It is notable that DEX treatment was required for GR occupancy and chromatin remodeling of the bICP0 E promoter in TG of calves during reactivation from latency. However, GR alone was sufficient for chromatin remodeling and bICP0 promoter activation in COS-7 cells. For example, previous studies demonstrated GR and KLF4 together activate the bICP0 E promoter independently of DEX in transient transfection studies performed in Neuro-2A cells (14). This seemingly contradictory result is due to inherent differences in TG neurons when compared to COS-7 cells. We suggest that chromatinization of viral genomes in latently infected sensory neurons is significantly different when compared to actively growing cells. Since LR gene products are the only viral gene abundantly expressed in latently infected neurons, it is possible LR gene products also directly or indirectly influence viral gene expression during latency.

It is well established that GR can function as a pioneer transcription factor because it interacts with heterochromatin and promotes chromatin remodeling, as reviewed in (60, 61). Based on our studies, it seems clear that GR-mediated activation of the IEtu1 promoter occurs via a ligand-dependent mechanism because DEX treatment is required for GR-mediated transactivation (12, 13), GR occupancy, promoter activity, and viral replication in COS-7 cells (Fig. 2). Additional studies revealed GR and cellular transcription factors, including KLF15 (13), E2F2 (62), Sp1, and/or Sp3 (46), cooperatively or synergistically activate IEtu1 promoter activity. Notably, the two GREs in the IEtu1 promoter are more than 1,000 bp from the TATA box, suggesting that secondary structures in the IEtu1 promoter lead to the juxtaposition of the GREs to the TATA box.

Although the bICP0 E promoter is cooperatively transactivated by GR, KLF4 (14), KLF15 (59), Sp1, and Sp3 (63), there are no consensus GREs, and the ½ GREs are not important for transactivation (14). GR and/or KLF4 occupy sequences in the first 328 bps and 328–638 of the bICP0 E promoter in transfection studies, and mutating G-rich (GGGCGG) and/or C-rich Sp1 binding sites (CCCGCC) significantly reduced transactivation by GR and KLF4, Sp1, or Sp3 (44, 63). Based on these studies, we suggest GR protein-protein interactions with KLF4, Sp1, Sp3, or unknown transcription factors recruit GR to the bICP0 E promoter via a tethering mechanism. Support for this premise comes from studies demonstrating GR interacts with several transcription factors and activates transcription via a tethering mechanism (64, 65). Finally, the bICP0 E promoter, but not the IEtu1 promoter, is activated via non-ligand-dependent mechanisms in cultured cells (66).

Notably, the BoHV-1 genome contains more than 100 potential GR-binding sites (12). Intergenic regions in the BoHV-1 genome that contain GREs or ½ GREs were inserted upstream of a simple promoter that drives luciferase expression, and we tested whether these intergenic sequences are activated by GR. An intergenic region in the viral DNA primase gene, UL52, is strongly transactivated by GR and KLF15 in the presence of DEX (12). Mutating one or both ½ GREs in the UL52 intergenic region significantly reduced GR and KLF15-mediated transactivation. A bICP4 intergenic region is also cooperatively transactivated by GR and KLF15, but the effect was not as dramatic as the UL52 fragment. Finally, GR and KLF4 had a modest transactivation on these intergenic regions, but DEX did not increase the transactivation of the ICP4 intergenic region. Since certain GREs in cellular chromosomes can stimulate promoter activity when they are 5,000–19,000 bp from the TATA box (67), GREs in intergenic regions of the BoHV-1 genome may play supportive roles in remodeling chromatin and activating viral gene expression during DEX-induced reactivation.

The finding that H3K9me3 occupancy of the bICP0 E and IEtu1 promoters was not significantly decreased after DEX treatment implied that only a small subset of BoHV-1 genomes undergo chromatin remodeling during early stages of reactivation from latency (Fig. 7). However, it is not clear whether a small percent of viral genomes in different latently infected TG neurons support DEX-induced reactivation or whether numerous viral genomes in a small subset of TG neuronal subtypes undergo successful reactivation from latency. Several distinct subtypes of TG neurons exist in mice, and A-5 positive sensory neurons are non-permissive for HSV-1 (68). If distinct sub-types of TG neurons exist in cattle, we suggest neuronal subtypes that express higher GR levels are more likely to support BoHV-1 reactivation from latency versus other neuronal subtypes that express little or no GR. Support for this prediction comes from two findings: (i) bICP0 +or VP16 +TG neurons are also GR+ (36, 37) and (ii) approximately 50% of sensory neurons in rat TG express GR, whereas other neurons do not express detectable GR (69).

As judged by immunohistochemistry studies, bICP0 and VP16 protein expression is detected prior to bICP4 (36–38). Although the bICP0 antibody may work better than the bICP4 antibody in immunohistochemistry studies, these antibodies detect their respective proteins efficiently in Western blots. KLF4, another pioneer factor, and GR cooperatively transactivate the bICP0 E promoter via a ligand-independent manner in transfected cells (14, 44), suggesting these two pioneer factors facilitate bICP0 expression during very early stages of reactivation when DEX is present in TG neurons at low concentrations. In contrast, GR and KLF4 do not efficiently transactivate the IEtu1 promoter (13). Finally, we suggest that neuron-specific splicing of the IEtu1 transcript may culminate in higher bICP0 expression than bICP4 expression in TG neurons during early stages of reactivation from latency. Hence, there are several scenarios by which bICP0 is expressed prior to bICP4 in TG neurons during reactivation from latency.

In addition to GR stimulating viral gene expression during early stages of reactivation from latency, increased corticosteroid levels interfere with immune responses and inflammation, reviewed in (70, 71). The finding that adding DEX to COS-7 cultures triggered viral infection (Fig. 2C) implied that impairing immune responses was important for viral replication. It is also possible that DEX activates other transcription factors. Studies designed to understand this intriguing observation are in progress. Since bICP0 and VP16 protein expression are detected in TG neurons by immunohistochemistry within 1 hour after DEX treatment of latently infected calves (36–38), we suggest the anti-inflammatory and impaired immune responses are important during later stages of reactivation from latency, including facilitating virus spread.

## MATERIALS AND METHODS

### Cells and viruses

African Green Monkey Kidney Fibroblasts transformed with a mutant strain of SV40 that expresses large T-antigen (COS-7, ATCC CRL-1651) were cultured in minimal essential medium (MEM) supplemented with 10% fetal bovine serum (FBS), L-glutamine (2 mM), penicillin (10 U/mL), and streptomycin (100 mg/mL) at 37°C with 5% CO_2_. Where indicated, 2% charcoal-stripped FBS was used in place of 10% FBS because stripped FBS contains lower corticosteroid levels that modulate GR and other transcription factors, as described previously (44, 72, 73). By filtering the FBS through active charcoal filters, many hydrophobic molecules, including corticosteroids, are selectively removed without altering amino acid, salt, or glucose levels. This reduces background GR activation in the absence of exogenously supplied DEX.

BoHV-1 Cooper strain was obtained from the National Veterinary Services Laboratory, Animal and Plant Health Inspection Services, Ames, Iowa, and propagated using Modlin-Darby bovine kidney cells (ATCC CCL-22). The Cooper strain is the primary BoHV-1 strain present in North America. Viral DNA was isolated from virions following ultracentrifugation using a sucrose cushion, as previously described (23, 74).

### Plasmids and primers

An expression plasmid containing the mouse glucocorticoid receptor α is referred to as GR-α throughout this manuscript. This construct was obtained from Dr. Joseph Cidlowski (NIH) and was previously used (44, 72, 73). Plasmids were prepared for transfection from bacterial stocks using alkaline lysis and two rounds of cesium chloride ultracentrifugation.

IEtu1 promoter primers used for this study are IEtu1 F (5′-TAGCCGCTCCATTCTCTC-3′) and IEtu1 R (5′-AAAAGTGGGGAAGCAGGG-3′) that yield a 218 bp fragment, as described previously (45). The bICP0 E promoter primers used for this study are EP-328 F (5′-GCCCCCCCCCAAAAACAC-3′) and EP-328R (5′-CAAGGCGAAACCCCCCAC-3′) that yield a 130 bp product, as described previously (44).

### Transfection

COS-7 cells (~3 x 10^6^) were seeded onto 100 mm dishes and cultured in MEM with 10% FBS. Two hours prior to transfection, cells were washed with PBS, and MEM containing 2% charcoal-stripped FBS was added. Cells were transfected with BoHV-1 DNA alone or with the GR expression plasmid using TransIT-X2 (Mirus MIR6005). An empty vector was added where necessary to maintain equal amounts of DNA in each reaction. At 24 hours post-transfection, the medium was replaced, and water-soluble DEX (10 uM, Sigma D2915) was added to denoted cultures. At 48 hours post-transfection, cells were washed with PBS, formaldehyde-crosslinked, and harvested in a passive lysis buffer (50 mM HEPES-KOH, pH 7.5; 140 mM NaCl; 1 mM EDTA; 1% Triton-X; 0.1% sodium deoxycholate; 0.1% SDS). Samples were processed with an ultrasonicator (QSonica) to generate DNA fragments ~ 500 nt in length and centrifuged to remove the cell debris. The lysate was used for chromatin immunoprecipitation as described below.

### Calf infection

TG for this study came from previously reported calf studies (40) that were stored at −80℃ since harvest. For these studies, male calves (~200 kg) were inoculated with 1 mL of a solution containing 1 × 10^7^ pfu/mL of the wt BoHV-1 in each nostril and ocular cavity for a total of 4 × 10^7^ pfu per animal. Sixty days following infection, latently infected calves were given an IV injection (jugular vein) of water-soluble DEX (100 mg, Sigma; D2915). Three hours following DEX treatment, calves were euthanized. TGs were harvested and placed on dry ice within 5 minutes of euthanasia. Frozen samples were then stored at −80℃. Latency control calves were euthanized and TG harvested as above, without receiving DEX treatment. For each ChIP experiment, three samples were taken from TG and processed independently as biological replicates.

### TG processing

Frozen TGs were finely sliced with a sterile razor blade using dry ice to maintain a cold temperature, and slices were subsequently fixed in formaldehyde. Individual 300 mg samples were ground to a fine powder in liquid nitrogen, using a mortar and pestle to break down the abundant extracellular matrix present in bovine TG. Each sample was suspended in 750 mL passive lysis buffer (50 mM HEPES-KOH, pH 7.5; 140 mM NaCl; 1 mM EDTA; 1% Triton-X; 0.1% sodium deoxycholate; 0.1% SDS) and briefly processed in an ultrasonicator (QSonica) to further break up the extracellular matrix and free individual cells. Samples were centrifuged at 1,000 × *g* to remove tissue and cell debris, and supernatants were transferred to new tubes. Brief sonication was used to generate chromatin fragments of ~500 nt in length. The lysate was centrifuged to remove the cell debris, and supernatants were used for ChIP as described below.

Bovine TG tissue contains significant extracellular structures with high levels of connective tissue that must be removed to prepare neurons for ChIP. Harvested TGs were immediately frozen at −80°C for storage. While keeping the tissue frozen using dry ice, 300 mg samples were thinly sliced from the whole TG, formaldehyde-crosslinked to form DNA-protein complexes, and subsequently ground into a fine powder using a liquid nitrogen-cooled mortar and pestle. Each sample was briefly homogenized in lysis buffer using an ultrasonicator. Extracellular material was removed by centrifugation, and the lysate was transferred to new tubes. Each sample was further sonicated to shear the DNA into ~500 nt fragments for further processing by ChIP.

### ChIP studies

The cell lysate was precleared in protein G agarose with salmon sperm DNA (Millipore 16-201). Following centrifugation, an input sample was removed, and the supernatant was split into an antibody sample and isotype control. ChIP was performed as previously described (44, 72, 73). In brief, samples were incubated overnight at 4℃ in RIPA buffer (50 mM Tris-HCl, pH 8.0; 150 mM NaCl; 2 mM EDTA; 1% NP-40; 0.5% sodium deoxycholate; 0.1% SDS) containing either GR (Cell Signaling 3660S), H3K9me3 (Abcam ab8898), H3K9acetyl (Abcam ab10812) antibodies or nonspecific rabbit IgG (Abcam ab171870) for the isotype controls. Antibody-protein-DNA complexes were precipitated using Protein A Dynabeads (Invitrogen 1000D), and washed five times: twice with a low salt wash buffer (20 mM Tris-HCl, pH 8.0; 150 mM NaCl; 2 mM EDTA; 1% Triton-X; 0.1% SDS), twice with a high salt wash buffer (20 mM Tris-HCl, pH 8.0; 500 mM NaCl; 2 mM EDTA; 1% Triton-X; 0.1% SDS), and once with a LiCl wash buffer (20 mM Tris-HCl, pH 8.0; 250 mM LiCl; 1 mM EDTA; 1% NP-40; 1% sodium deoxycholate). Protein-DNA complexes were eluted from the beads (1% SDS; 100 mM NaHCO_3_) at 30℃ for 30 minutes, and crosslinking was reversed at 65℃ overnight, with RNase A and proteinase K added to each sample. DNA was prepared using phenol-chloroform and amplified by PCR. Individual bands were separated on an agarose gel, visualized by UV with ethidium bromide (BioRad ChemiDoc MP), and quantified using Image Lab software (BioRad).

### Luciferase assay

COS-7 cells were seeded (8 × 10^5^) onto 60 mm dishes containing MEM supplemented with 10% FBS 24 hours prior to transfection. Two hours prior to transfection, cells were washed, and MEM supplemented with 2% charcoal-stripped FBS was added. Cells were transfected with either the IEtu1 promoter construct or bICP0 E promoter construct cloned into the pGL4.24 plasmid driving the expression of firefly luciferase using TransIT-X2 transfection reagent (Mirus MIR6005), with a pGL4.24 plasmid encoding a Renilla luciferase gene acting as a transfection control. Certain samples were additionally cotransfected with the GR-α expression plasmid, while the empty expression vector was added to other samples to maintain equal DNA concentrations. At 24 hours post-transfection, the denoted samples were treated with water-soluble DEX (10 uM, Sigma D2915). At 48 hours post-transfection, samples were harvested and processed using a commercially available dual luciferase assay kit (Promega, E1910). Luciferase activity was measured using a GloMax 20/20 luminometer (Promega, E5331).
